# Hyperthermic intraperitoneal chemoperfusion with high dose oxaliplatin: Influence of perfusion temperature on postoperative outcome and survival

**DOI:** 10.12688/f1000research.2-179.v2

**Published:** 2015-10-16

**Authors:** Johanna Verhulst

**Affiliations:** 1 Independent Researcher, Karel Oomsstraat 57, Antwerpen, 2018, Belgium

**Keywords:** HIPEC, cancer therapeutics, survival rates

## Abstract

**Introduction** **:** Hyperthermic intraperitoneal chemotherapy (HIPEC) is becoming a standard therapy in the treatment of peritoneal carcinomatosis (PC). Compared to systemic chemotherapy, HIPEC improves survival in patients with PC. This therapy has high morbidity rates (up to 41%). In vitro it has been demonstrated that hyperthermia has a toxic effect on malign cells. However, hyperthermia also affects normal tissue. To my knowledge, any additional effect of hyperthermia combined with chemotherapy has never been demonstrated in a clinical setting. In this study, the effects of hyperthermia on outcome and survival were analyzed.

**Methods** **:** Patients with PC from any origin who were treated with HIPEC were included in this retrospective, non-randomized study. Data on patient characteristics, tumor characteristics, features of the surgery and postoperative complications were extracted from patient files. Models predicting time to removal of nasogastric tube (TRNT), post-operative major complications, the occurrence of anastomotic leaks and post-operative survival were built, using negative binomial regression, logistic regression or Cox proportional hazards regression as appropriate.

**Results**: 138 patients treated with HIPEC were included. Maximal temperature during the operation was not statistically significantly associated with anastomotic leaks or post-operative major complications. Maximal temperature during the operation was negatively associated with post-operative survival (P=0.01).

**Conclusion** **:** The results suggest that hyperthermia may negatively affect survival in patients who are treated with HIPEC for PC of various origins. This study has the classical limitations of a retrospective study. Therefore, randomized trials are required to confirm the results.

## Declaration

The research data that were used by the author are part of a study supported by the Special Research Fund of Ghent University (BOF11/24J/072). The responsibility of the study supervisor was limited to the data collection and processing. The supervisor dissociates himself from any conclusions on this data made by the author.

## Introduction

Peritoneal carcinomatosis (PC) occurs in 5% of patients with colorectal carcinoma and in patients with FIGO (International Federation of Gynecology and Obstetrics) stage III and IV ovarian cancer ^1^. PC can occur synchronous with the primary tumor or as a relapse (metachronous). Survival rates in patients with PC are rather poor. The median survival with standard chemotherapy is 50 and 23 months for PC of ovarian and colorectal origin, respectively ^2, 3^. When treated with hyperthermic intraperitoneal chemotherapy (HIPEC), survival rates of patients with PC of ovarian and colorectal origin increased to 66 and 30 months, respectively ^2, 4^.

There is an increasing interest in the use of locoregional antineoplastic drug therapy in patients with PC. The benefit of intraperitoneal chemotherapy arises from the existence of a peritoneal-plasma barrier. This barrier allows the local administration of higher doses of chemotherapy while minimizing systemic side effects ^5^. Cytotoxic drugs penetrate only a few millimeters into tumor tissue ^6^. To improve penetration, HIPEC is combined with cytoreductive surgery, where the tumor mass is decreased as much as possible before the administration of chemotherapy.

Oxaliplatin is recognized as a standard adjuvant treatment in colorectal cancer ^7^. Promising results were also demonstrated in ovarian cancer, gastric cancer and malignant mesothelioma ^8– 10^. Oxaliplatin is rapidly absorbed intracellularly, as a result of its lipophilic structure ^11^. Combined, these features make oxaliplatin a logical choice for local administration.

Hyperthermic perfusions are used because hyperthermia stimulates apoptosis in tumor cells ^12– 14^. Recently it was demonstrated that hyperthermia increases the peritoneal oxaliplatin concentration while reducing systemic absorption ^15^. However hyperthermia also induces apoptosis in normal cells ^14^ and affects the healing of anastomosis ^16– 18^. Furthermore, it was demonstrated in a rat model of PC of colorectal origin that hyperthermia did not increase survival compared to normothermic intraperitoneal treatment ^19^. To my knowledge, any additional effect of hyperthermia combined with intraperitoneal chemotherapy compared to intraperitoneal chemotherapy alone has not been demonstrated.

Although in a recent trial the preoperative level of functioning was reached three to six months after surgery ^20^, the morbidity rates described in patients after HIPEC are rather high (up to 41%) ^21^. The role of hyperthermia in the improved survival is not clear, and it is possible that the morbidity may be (partially) a result of the hyperthermia.

The aim of this study was to identify the impact of the temperature of the perfusate on post-operative ileus, major post-operative complications, the occurrence of anastomotic leaks and post-operative survival.

## Materials and methods

This is a retrospective, non-randomized study. By the retrospective and anonymised nature of the study, no informed consent of the patients was required. The study included patients from one university hospital. All patients that presented with resectable PC from any origin were eligible for inclusion. Patients with primary PC were included, as well as patients with metachronous PC. Electronic patient files were reviewed and the following data were extracted: age at the time of the operation, gender, body mass index (BMI), duration of anesthesia, time to removal of nasogastric tube (TRNT, measured from the day of operation), duration of stay in intensive care unit (ICU), duration of total hospital stay, 30-days mortality, post-operative complications, maximal perfusate temperature (Tmax), and area under the temperature curve (AUC) as a measure of total temperature. Analyses of biochemistry and cell count were carried out on blood samples taken on the last day before and the first day after HIPEC. White blood cell count, aspartate aminotransferase (AST), alanine aminotransferase (ALT) and gamma-glutamyltransferase (γ- *GT*) were registered. TRNT was used as a measurement of the duration of post-operative ileus.

The temperature of the perfusate was measured in three locations: left and right in the upper abdomen, and in the pelvis. AUC was calculated separately for each registration location with a data summary model for repeated measures (baseline = 0) in Medcalc™ 12.5.0 (MedCalc Software, Acacialaan 22, B-8400 Ostend, Belgium.) The mean AUC over the three locations was used in this study. The unit of AUC is °C*minute.

The full dataset is provided in the accompanying Data File. Blanks in these tables represent missing data.

### Hyperthermic perfusion

Patients were placed in modified Lloyd Davies position and the upper body covered with a heating blanket (Bair Hugger, Arizant Healthcare Inc., Eden Prairie, MN, USA). Cytoreductive surgery aimed to remove all resectable implants of tumor while preserving the patient’s quality of life. Following verification of resectability and absence of undetected metastatic disease, the entire colon was mobilized starting from the ileocolic region working towards the left. The major omentum was removed en bloc with the affected colon whenever it was involved in the disease process. The spleen, or pancreatic tail were included in the specimen when affected by cancer. A peritonectomy of the diaphragm was performed according to a previously described method ^22^. When required, the tendinous part of the diaphragm was partially resected. Following the resections in the upper abdomen, tumor tissue was removed from the pelvis. After that, the serosal surfaces covering the small bowel and mesentery were cleared from tumor tissue by a combination of tumorectomy, wedge resection, or segmental resection as required. At least 150 cm of small bowel had to be preserved. An open abdomen method was used for the administration of the intraperitoneal chemoperfusion, as described previously ^23^. The skin was sutured to a retractor frame placed over the abdomen. A plastic hood was positioned over the frame in order to allow the evacuation of vapor escaping from the abdominal cavity. Two Tenkhoftype inflow drains and three multiperforated outflow drains connected to a roller pump were used for chemoperfusion. The drains were placed in the pelvis, right upper abdomen and left upper abdomen. A heat exchanger was placed along the drains in order to maintain the required temperature. Hypothermia (34°C) was maintained prior to the start of chemoperfusion. Central temperature was monitored with an esophageal temperature probe. Abdominal temperature was monitored by means of three thermocouple probes placed left and right in the upper abdomen, and in the pelvis.

Prior to chemoperfusion, intravenous chemotherapy, consisting of folate 20 mg/m ^2^ followed by a 5-fluorouracil 400 mg/m ^2^ in 250 ml of saline perfusion over 1 hour, was administered to non-ovarian cancer patients according to standard procedures. Oxaliplatin (460 mg/m ^2^) was added to the perfusion circuit when the abdominal temperature reached the set temperature. The duration of the chemoperfusion was 30 minutes. The abdominal cavity was not washed, in order to retain the efficacy of remaining drug. After the chemoperfusion, the abdomen was closed in layers.

### Statistical analysis

The primary end-point of this study was overall survival. Overall survival was measured from the day of surgery till death. Patients who were alive at the last contact moment were censored at the date of last contact.

The secondary end-points were major complications, the occurrence of anastomotic leaks and TRNT. TRNT was a proxy variable for post-operative ileus. Univariate relations between Tmax or AUC and stay at ICU, total hospital stay, post-operative white blood cell count, AST, ALT, γ- *GT* and TRNT were explored by means of linear regression or negative binomial regression, as appropriate. Univariate relations between Tmax or AUC and overall survival were explored by means of Cox-regression.

A negative binomial regression model predicting TRNT was built using Tmax, AUC, sex, age at the time of the operation, operation time, stay at ICU, post-operative white blood cell count, AST, ALT and γ- *GT* as independent variables. The parameters “major complications” and “anastomotic leaks” are binary variables; therefore a logistic regression model was built to predict these outcomes, including Tmax, AUC, sex, age at the time of the operation, BMI, post-operative white blood cell count, AST, ALT and γ- *GT*, operation time and number of anastomoses as independent variables. A Cox proportional hazards model predicting overall survival was built using Tmax, AUC, sex, age at the time of the operation, operation time, number of anastomoses, tumor type, stay at ICU, total hospital stay, post-operative white blood cell count, AST, ALT and γ- *GT* as independent variables. Backwards stepwise selection was used for model building. Statistical significance was assumed when P<0.05.

## Results

**Figure 1.**
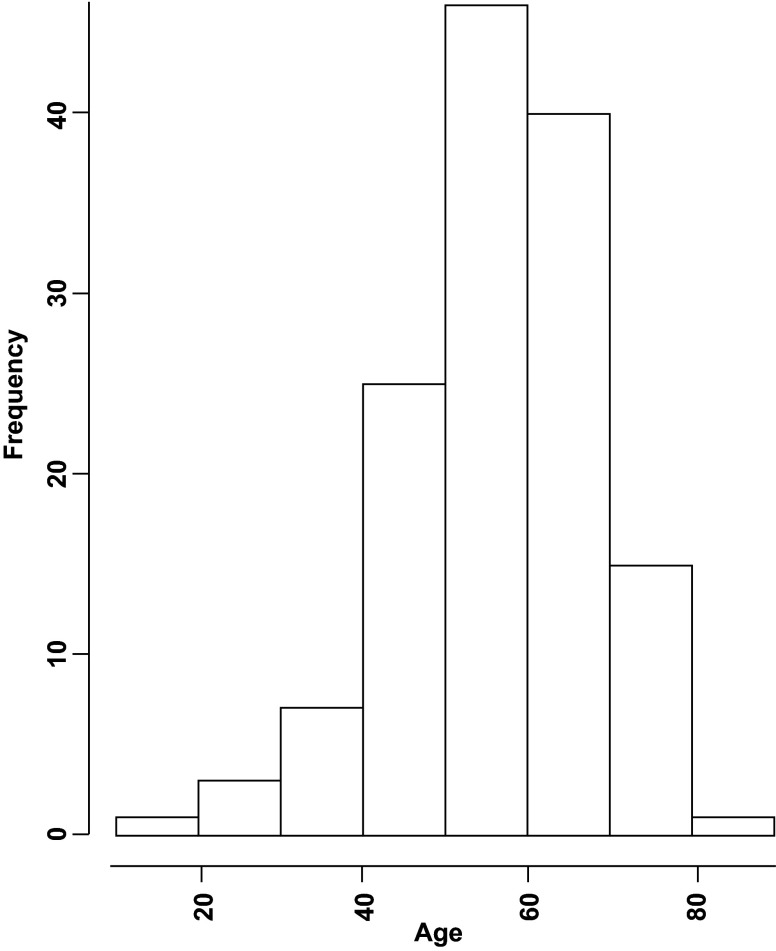
Distribution of age (years). Histogram of frequencies of ages of the included patients.

From July 2005 until February 2011, 138 patients were treated with oxaliplatin-based HIPEC in a tertiary center. Demographic data are illustrated in Table 1. Mean age was 59 years, ranging from 17 to 82 years ( Figure 1). Forty-four percent of the patients were males. Nearly 60% of the patients presented with PC originating from colorectal cancer. Ovarian cancer and pseudomyxoma peritoneii were the second and third most frequent cause of the PC, respectively.

**Table 1.** Demographic details of 138 patients treated with oxaliplatin based HIPEC (*:minimum-maximum).

Age (mean, years)	59	17–82*
Male/Female (%)	44/56	
BMI (mean)	24.1	16.8–34.1*
Synchronous/Metachronous disease (%)	35/65	
Histology (%)		
Colorectal	58	
Ovarian	12	
Pseudomyxoma peritoneii	11	
Other	19	

**Figure 2.**
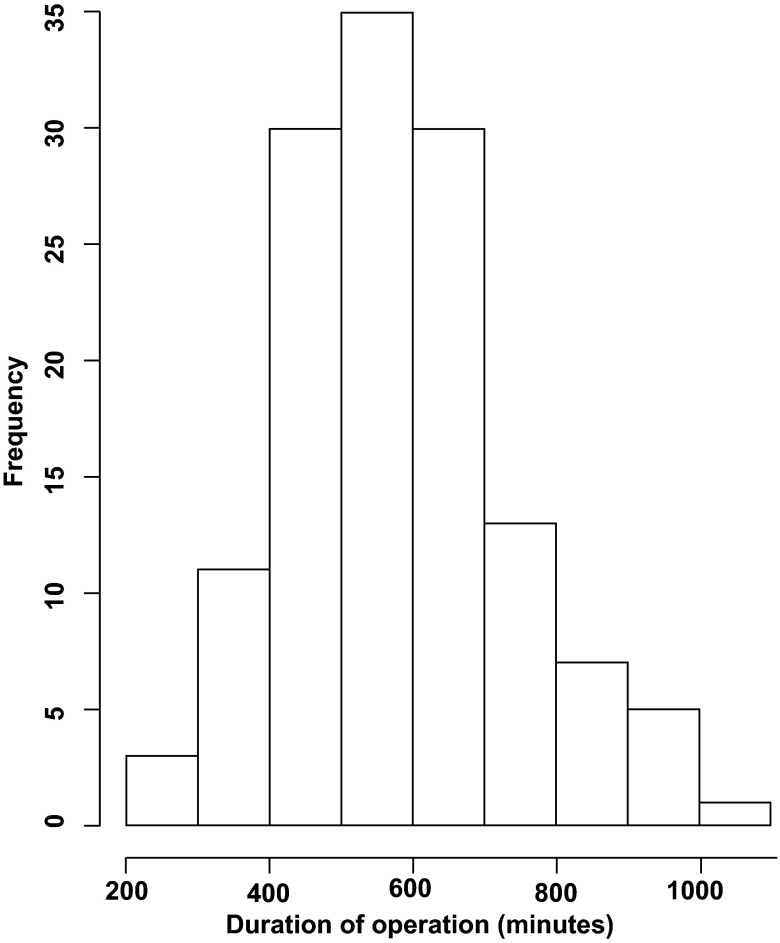
Distribution of duration of aneasthesia (minutes). Histogram of frequencies of the duration of aneasthesia in the included patients.

**Figure 3.**
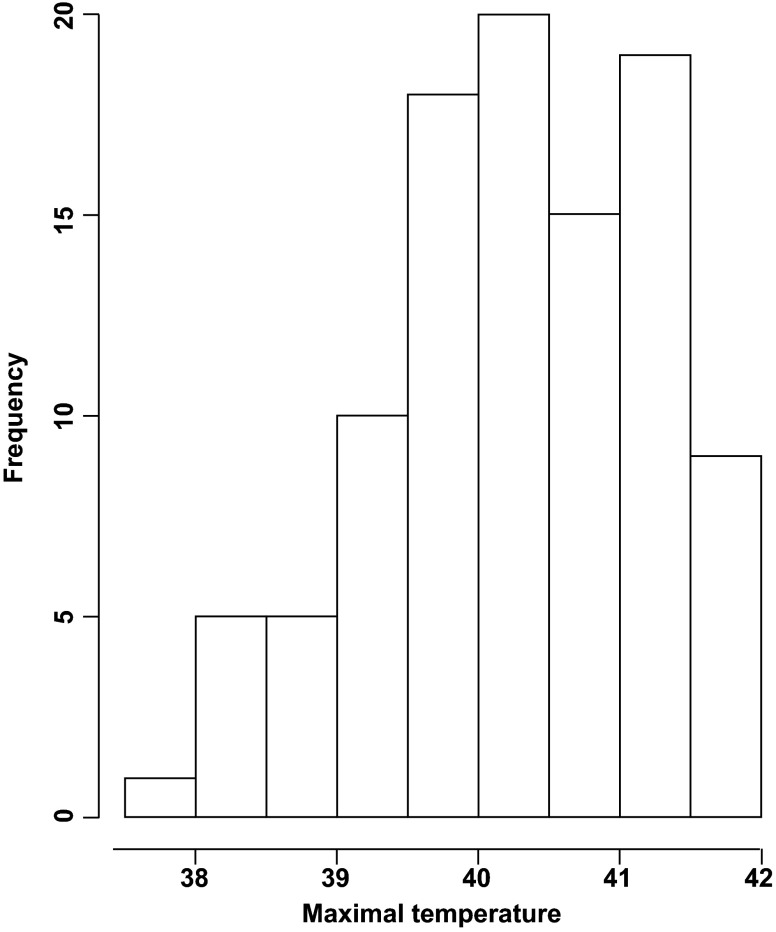
Distribution of maximal temperature (Tmax) (°C). Histogram of frequencies of maximal temperature of the included patients.

**Figure 4.**
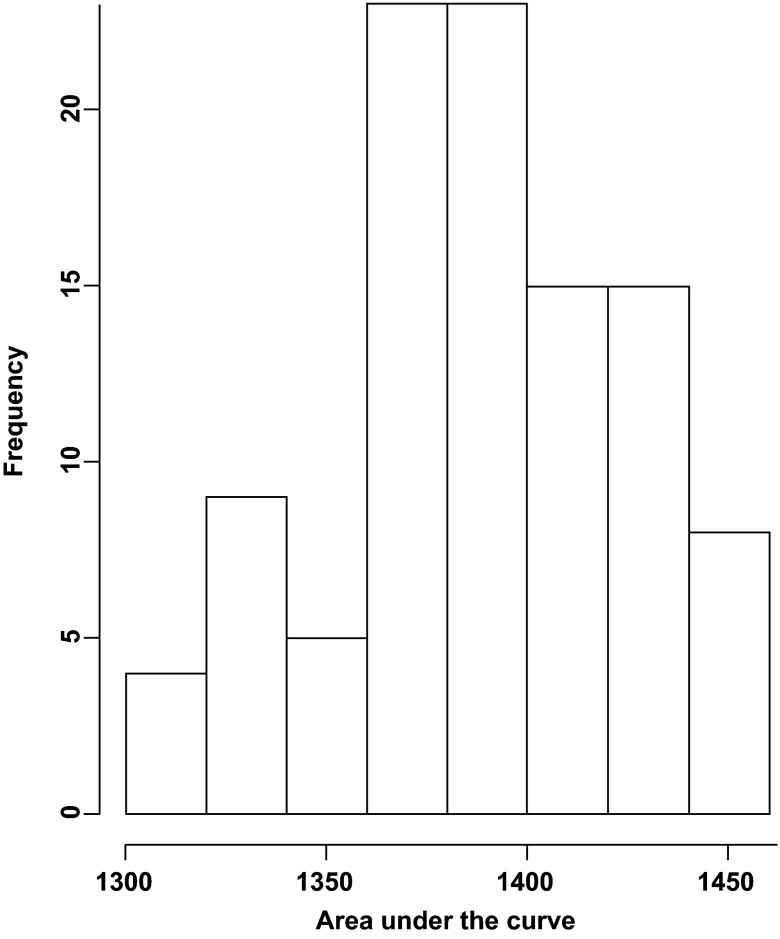
Distribution of area under the curve (°C*minutes). Histogram of frequencies of the AUC of the included patients.

Details of the surgery are illustrated in Table 2. The mean anesthesia time was nearly 10 h, ranging from 4 to 18 h and with a standard deviation of 2.8 h ( Figure 2). On average, the maximal temperature was 40.5°C and the area under the temperature curve was 1340.63°C*minute ( Figure 3 and Figure 4).

**Table 2.** Details of surgery (Mean and Standard deviation).

Operating time (minutes)*	579	166
Number of anastomoses (%)		
	0	35.5
	1	30.4
	2	25.4
	3	5.1
	4	2.2
	5	1.4
Peak temperature (°C)*	40.510	1.143
AUC*	1340.63	91.63

Outcome of surgery is summarized in Table 3. Two patients (1.4%) died within 30 days after the operation. Due to the small number of events the influence of AUC and maximal temperature on 30 days mortality could not be examined. Twenty-six patients needed reoperation. Reasons for reoperation were anastomotic leak (fourteen patients), intra-abdominal bleeding (five patients), perforation of the stomach (one patient), subobstruction (one patient), wound infection (one patient), bladder leak (one patient), and abdominal collection (three patients). In one patient, scald injuries were found during reoperation. Eighty-nine patients had at least one anastomosis, resulting in 155 anastomoses. Anastomotic leak occurred in sixteen patients. However, neither AUC nor Tmax was related to anastomotic leaks (P=0.68 and P=0.67, respectively) or major complications (P=0.50 and P=0.20, respectively).

**Table 3.** Outcome of surgery (* % of patients with at least one anastomosis ** Data represent median and range).

30-day mortality n (%)	2 (1.4)
Major complications n (%)	38 (27.5)
Anastomotic leaks n (%)	16 (18.0)*
Reoperation rate n (%)	26 (18.8)
Median ICU stay (days)	2 (0–87)**
Median hospital stay (days)	18 (3–169)**

A logistic regression model assessing the relation between several predictors and the occurrence of anastomotic leaks was fitted ( Table 4). Longer operation time, a high number of anastomoses and post-operative leukocyte count were associated with the occurrence of leaks. Two variables describing the temperature during the operation were included in the model: Tmax and AUC. Both were close to significance. However, the effects of these variables were going in opposite directions: increasing AUC was associated with the occurrence of leaks, while increasing Tmax was associated with no leaks.

**Table 4.** Model predicting the odds ratio for no leaks versus leaks.

Variable	Estimate	SE	95% CI	P	OR
Intercept	0.8	15.9			
Number of anastomoses	-0.7	0.3	-1.4 to -0.1	0.03	0.50
AUC	-0.09	0.05	-0.18 to 0.01	0.07	0.91
Tmax	3.1	1.7	-0.1 to 6.4	0.06	22.20
Operation time	-0.0050	0.0025	-0.0098 to -0.0002	0.04	0.995
Post-operative leukocyte count	-0.2	0.1	-0.3 to -0.03	0.02	0.82

The number of anastomoses and the total operation time were associated with the occurrence of major complications ( Table 5). Tmax and AUC were not related to the occurrence of major complications.

**Table 5.** Model predicting the odds ratio for no major complications versus major complications.

Parameter	Estimate	SE	95% CI	P	OR
Intercept	3.2	0.8	1.6 to 4.9	<0.01	
Number of anastomoses	-0.30	0.17	-0.64 to 0.05	0.09	0.74
Operation time	-0.003	0.001	-0.006 to -0.001	0.01	0.997

**Figure 5.**
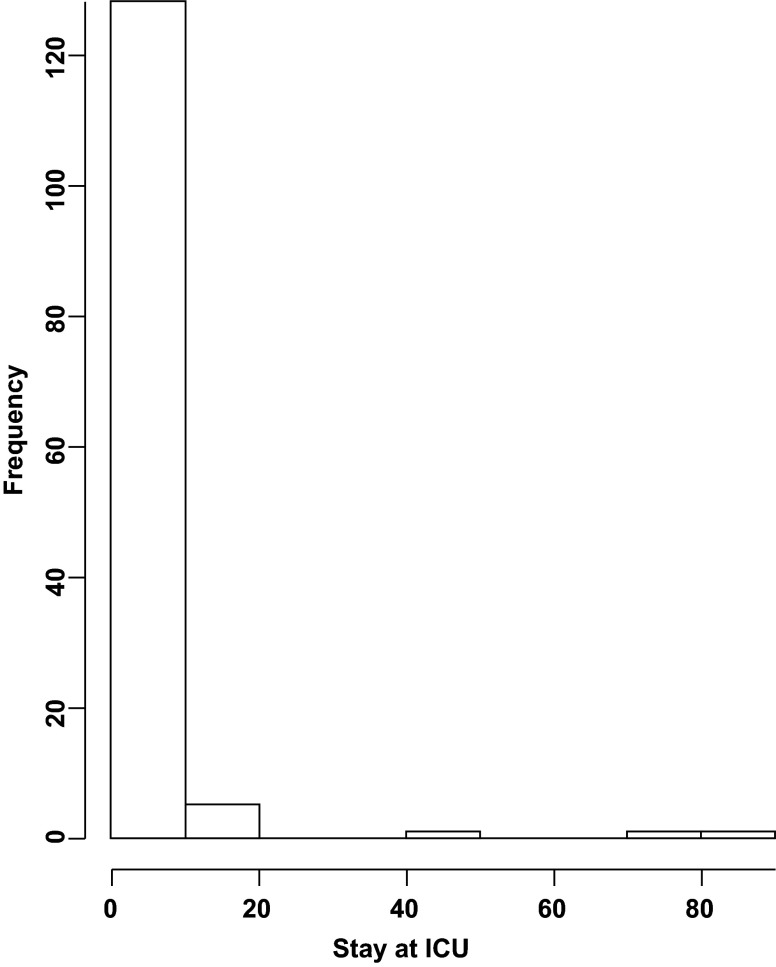
Distribution of stay at ICU (days). Histogram of frequencies of stay at ICU in the included patients.

**Figure 6.**
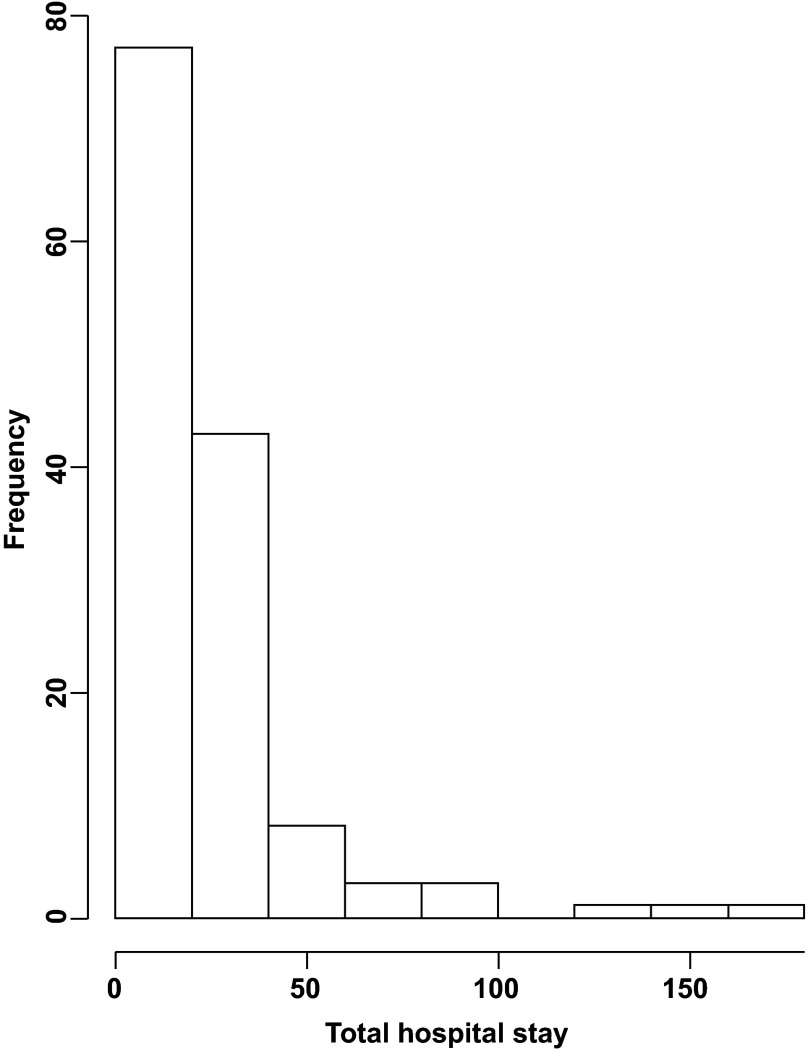
Distribution of total hospital stay (days). Histogram of frequencies of duration of hospitalization in the included patients.

**Figure 7.**
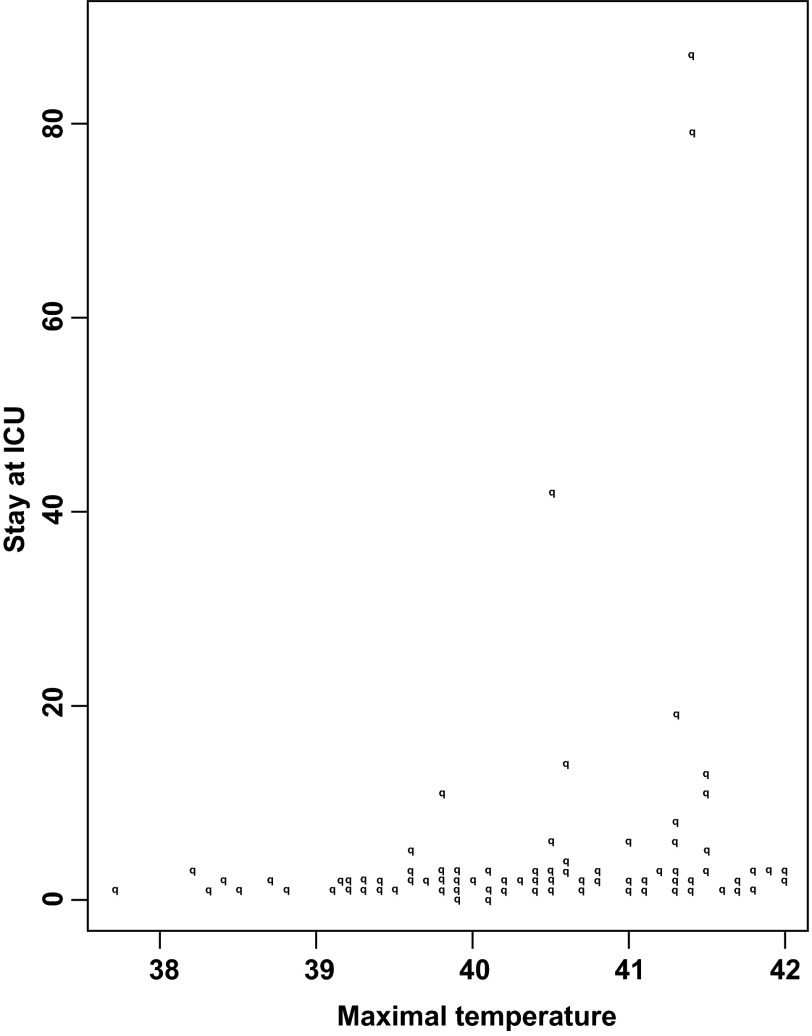
Stay at ICU (days) versus maximal temperature (°C). Scatterplot showing the relation between stay at ICU and Tmax.

**Figure 8.**
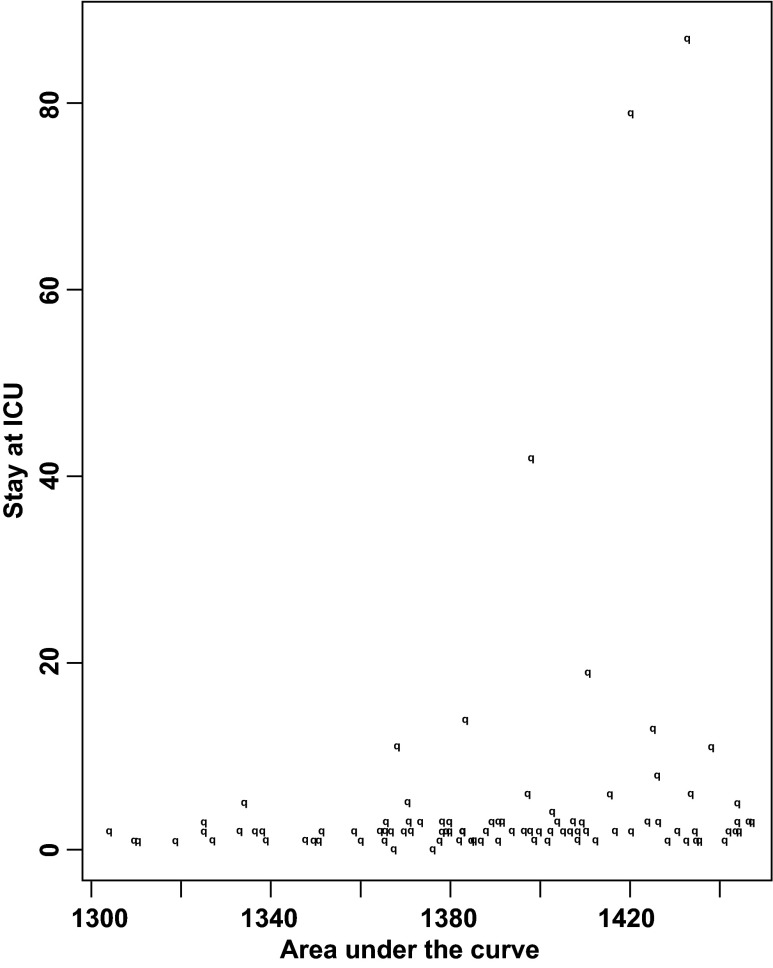
Stay at ICU (days) versus area under the curve (°C*minutes). Scatterplot showing the relation between stay at ICU and AUC.

**Figure 9.**
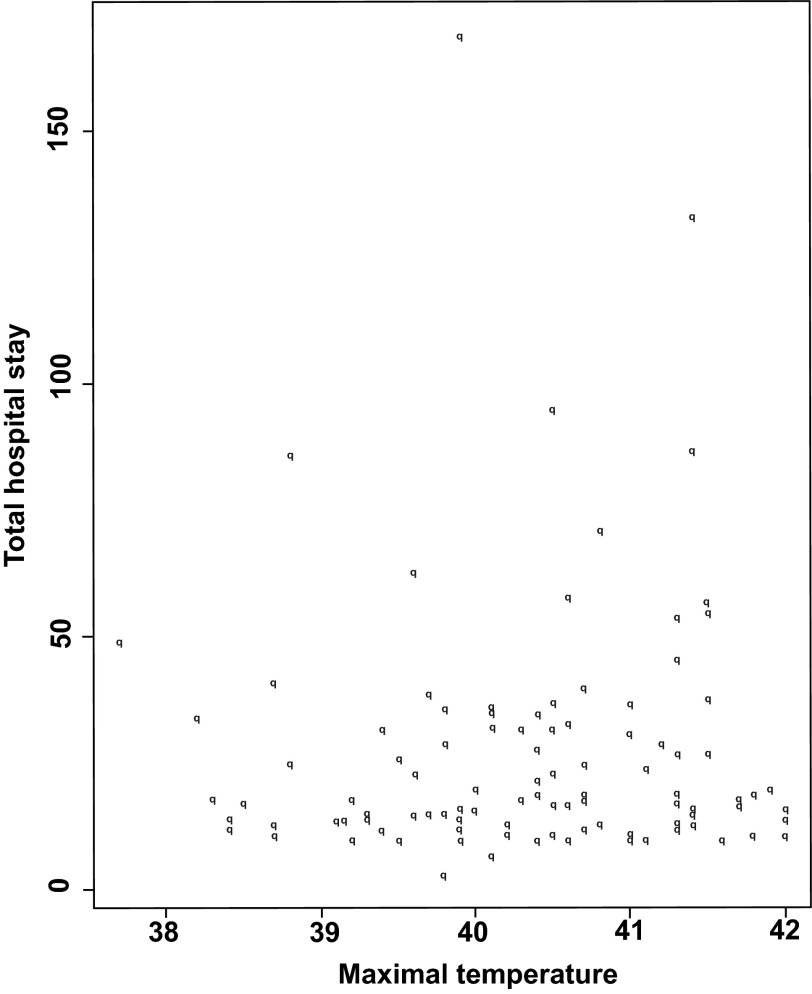
Total hospital stay (days) versus maximal temperature (°C). Scatterplot showing the relation between total hospitalization duration and Tmax.

**Figure 10.**
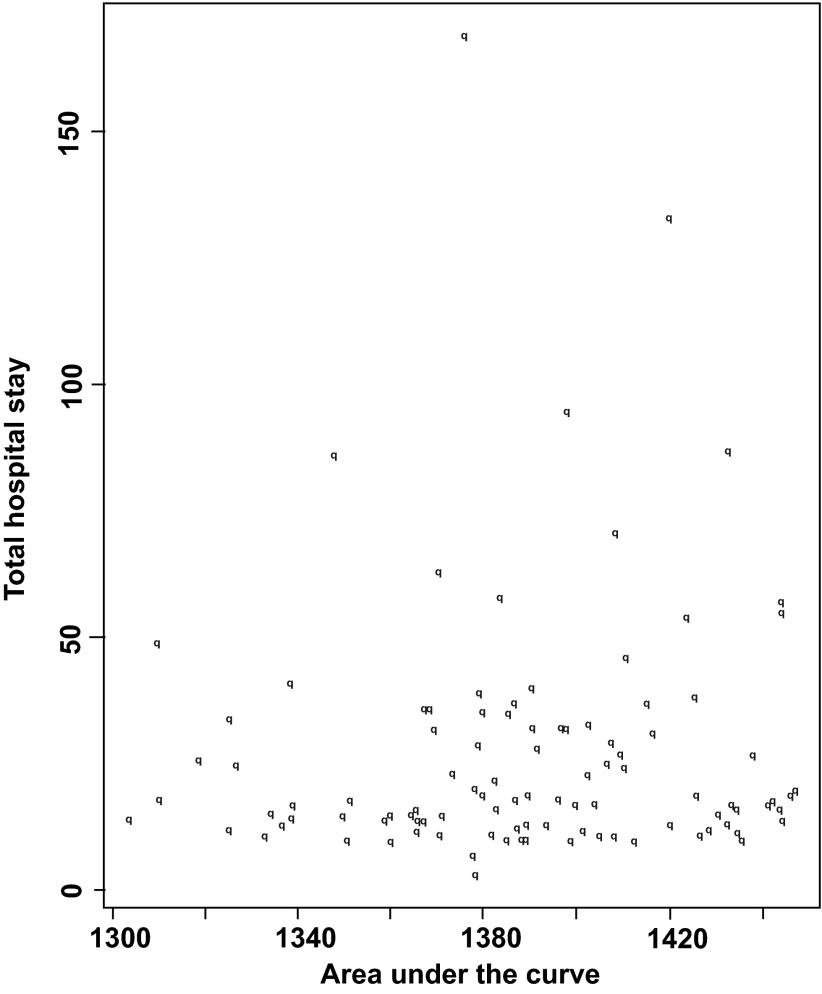
Total hospital stay (days) versus area under the curve (°C*minutes). Scatterplot showing the relation between hospitalization duration and AUC.

On average, patients stayed 4 days in the ICU (median 2, range 1 to 87, Figure 5). The total hospital stay was 27 days on average (median 18, range 3 to 169, Figure 6). The relationship between stay at ICU and Tmax or AUC is illustrated in Figure 7 and Figure 8, respectively. From these figures, it seems that patients with an extremely long stay at the ICU were treated at higher temperatures. In terms of the total hospital stay, there were fewer outliers and the duration was more evenly spread as a function of Tmax and AUC ( Figure 9 and Figure 10).

**Figure 11.**
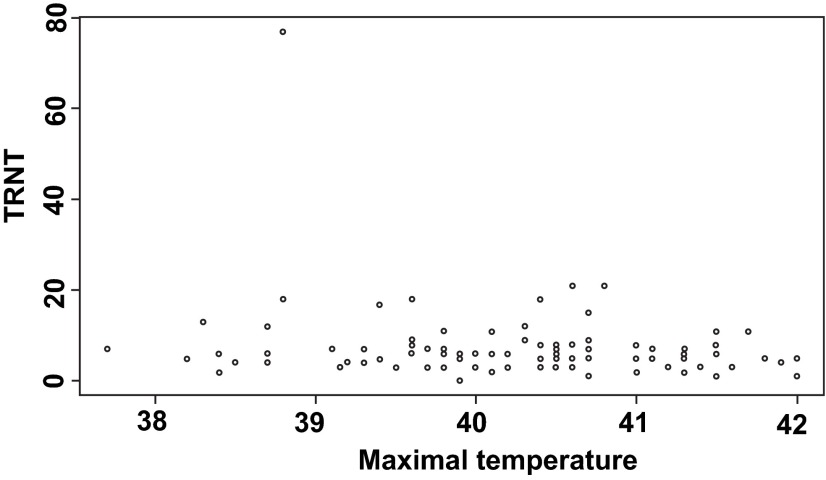
Time to removal of nasogastric tube (TRNT) (days) versus Tmax (°C). Scatterplot showing the relation between TRNT and Tmax.

**Figure 12.**
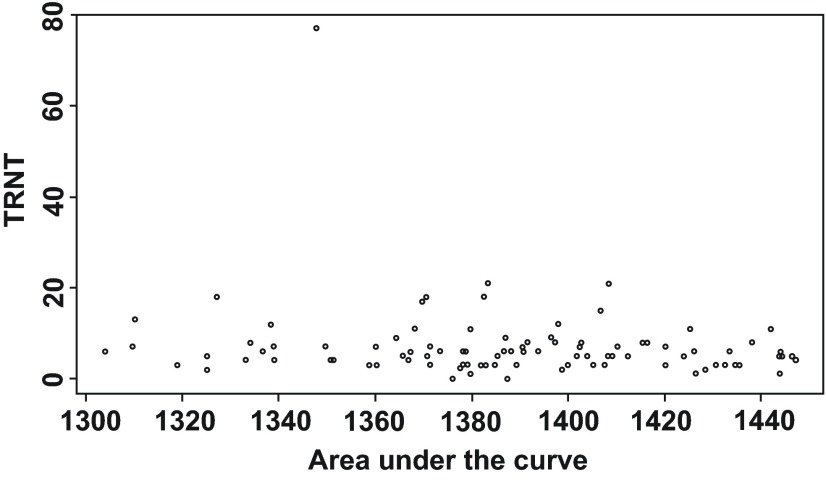
Time to removal of nasogastric tube (TRNT) (days) versus area under the curve (°C*minutes). Scatterplot showing the relation between TRNT and AUC.

On average, the nasogastric tube was removed after 7 days (median 5, range 0 to 77). The time to removal of the nasogastric tube seemed to remain constant with increasing temperature ( Figure 11 and Figure 12). A model predicting the time to removal of nasogastric tube was fitted ( Table 6). Four of the assessed variables turned out to be significantly related to TRNT: sex, Tmax, operation time and post-operative leukocyte count. On average, removal of the nasogastric tube was sooner after the operation in females than in males. With increasing maximal temperature, the expected TRNT decreased. Increasing post-operative leukocyte count and operation time was associated with an increased expected TRNT.

**Table 6.** Model predicting the logarithm of time to removal of nasogastric tube (TRNT) (dispersion parameter 0.3 (95% CI 0.2 to 0.5), scaled deviance 94.9 on 88 degrees of freedom).

Variable	Estimate	SE	95% CI	P
Intercept	10.2	3.1	4.2 to 16.2	<0.001
Sex	-0.4	0.1	-0.6 to -0.1	0.02
Tmax	-0.2	0.08	-0.4 to -0.1	<0.01
Operation time	0.001	0.0005	0.0003 to 0.0020	0.01
Post-operative leukocyte count	0.03	0.01	0.008 to 0.061	0.01

Median survival was 23 and 27 months in patients with PC from colorectal and ovarian origin, respectively ( Table 7). In univariate analysis Tmax was significantly associated with hazard of death (P=0.042), while AUC was not significantly associated to this hazard (P=1.117) ( Table 8).

**Table 7.** Survival (in months).

Primary tumor	Mean	95% CI	Median	95% CI
Colorectal	29	22–36	23	15–31
Ovarian	30	16–44	27	0–55

**Table 8.** Cox regression.

	P	Exp(B)	95,.0% CI for Exp(B)
AUC	0.117	0.997	0.994–1.001
Tmax	0.042	1.306	1.010–1.688

**Figure 13.**
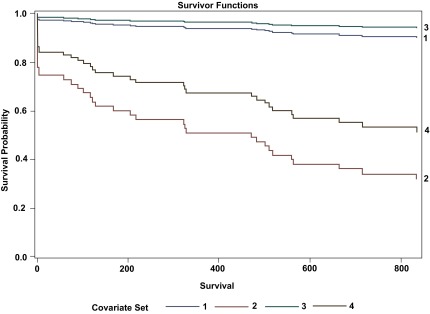
Estimated survival (days) curves for patients with PC from colorectal origin. 1: males, 37°C; 2: males, 42°C; 3: females, 37°C; 4: females, 42°C. All at 579 minutes operation time (mean).

**Figure 14.**
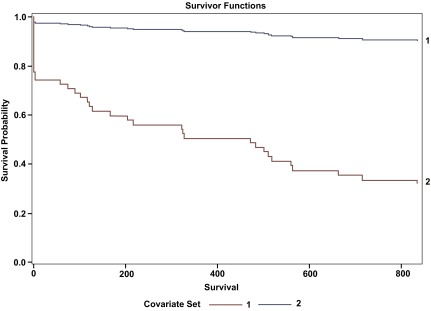
Estimated survival (days) curves for patients with PC from ovarian origin. 1: 37°C; 2: 42°C. All at 579 minutes operation time (mean).

A model predicting the hazard of death was built. Four of the considered variables turned out to be significantly related to this hazard: tumor type, sex, maximal temperature and operation time ( Table 9). The expected hazard ratio for an increase of 1°C in maximal temperature was 1.6, with the other variables in the model kept at fixed values. The predicted survival curves from this model are presented in Figure 13 and Figure 14.

**Table 9.** Model predicting the logarithm of the hazard of death (HR: Hazard ratio; SE: Standard error).

Variable		Estimate	SE	P	HR	95% CI
Tumor type	Other	1.1	0.4	<0.01	3.0	1.3 to 6.6
	PMP	-0.9	0.6	0.2	0.4	0.1 to 1.5
	Ovarian	0.6	0.5	0.3	1.7	0.7 to 4.5
Sex		-0.5	0.4	0.1	0.6	0.3 to 1.2
Tmax		0.5	0.2	0.01	1.6	1.1 to 2.3
Operation time		0.003	0.001	<0.01	1.003	1.001 to 1.006

A model including AUC instead of Tmax showed similar results. In this model the expected hazard ratio increased with 1.01 (P=0.01) for an increase of one unit in AUC, with other variables kept fixed. (Units of AUC are minutes °C).

**Figure 15.**
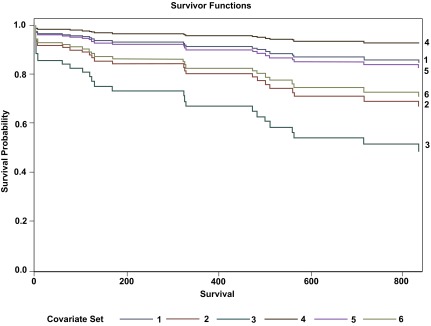
Estimated survival (days) curves for patients with PC from colorectal origin. 1: males, 37°C; 2: males, 40°C; 3: males, 42°C; 4: females, 37°C; 5: females, 40°C; 6: females, 42°C. All at 579 minutes operation time (mean) and 9.9 lactate mmol/l (mean).

**Figure 16.**
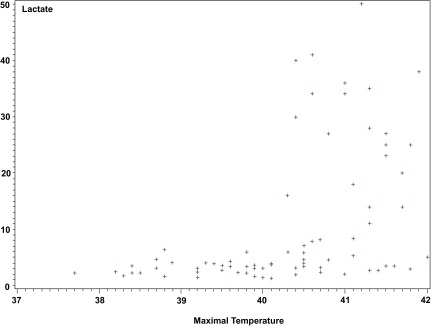
Blood lactate concentration (mmol/l) versus maximal temperature (°C). Scatterplot showing the relation between blood lactate concentration and Tmax.

The following analysis was not initially planned. An additional model predicting the hazard of death was built to evaluate the whether or not blood concentration of sodium, potassium, glucose, lactate, and pO _2_ or pCO _2_ during the operation were significantly related to post-operative survival. The variables from the first model were included as well. Tumor type, operation time, sex and lactate concentration in the blood were significantly associated with hazard of death ( Table 10). The predicted survival curves from this model are presented in Figure 15. Tmax was not significantly associated with the hazard of death after blood lactate concentration was included in the model. This is probably due to high correlation between Tmax and lactate concentration ( Figure 16).

**Table 10.** Model predicting the logarithm of the hazard of death, after inclusion of metabolic variables (HR: Hazard ratio; SE: Standard error).

Variable		Estimate	SE	P	HR	95% CI
Tumor type	Other	1.4	0.5	<0.01	4.2	1.7 to 10.2
	PMP	-0.4	0.6	0.5	0.7	0.2 to 2.4
	Ovarian	0.6	0.6	0.3	1.9	0.6 to 5.9
Lactate		0.04	0.01	<0.01	1.04	1.02 to 1.1
Sex		-0.7	0.4	0.08	0.5	0.2 to 1.1
Operation time		0.003	0.001	0.01	1.003	1.001 to 1.006

**Figure 17.**
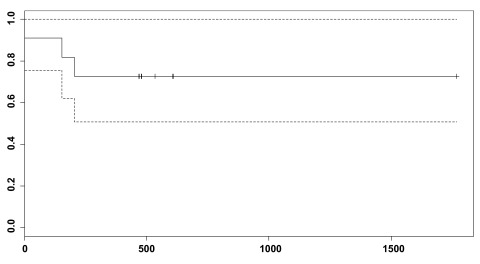
Actual survival curve for 11 patients with peritoneal carcinomatosis from any origin who were treated at a maximal temperature lower than 39°C.

**Figure 18.**
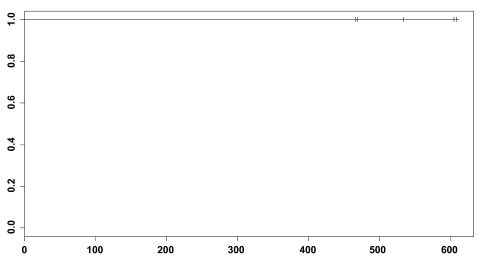
Actual survival curve for 5 patients with peritoneal carcinomatosis from colorectal origin who were treated at a maximal temperature lower than 39°C.

The survival curves in Figure 13– Figure 15 are predicted from the models. Actual survival curves for patients treated at a maximal temperature lower than 39°C are shown in Figure 17 and Figure 18.

Influence of perfusion temperature during hyperthermic intraperitoneal chemoperfusion on post-operative outcome and survival Data shows clinical and biochemical features in patients with peritoneal carcinomatosis from different primary tumors. A list of abbreviations is included in the second file. Click here for additional data file. Copyright: © 2015 Verhulst J 2015 Data associated with the article are available under the terms of the Creative Commons Zero "No rights reserved" data waiver (CC0 1.0 Public domain dedication).

## Discussion

In selected patients, cytoreductive surgery combined with hyperthermic intraperitoneal chemoperfusion (HIPEC) results in a better survival compared with systemic chemotherapy ^24^. Major complications occur frequently in the post-operative period. Hyperthermia is a possible risk factor for reduced anastomotic healing ^16– 18^. There is currently no evidence for better survival in patients treated with hyperthermic chemoperfusion compared to normothermic chemoperfusion. The aim of this study was to analyze the effects of hyperthermia on post-operative survival and on the post-operative complications.

The data presented here show a statistically significant association between increasing temperature during the perfusion and decreasing post-operative survival. More precisely, the expected hazard ratio is 1.6 times as large for an increase of 1 degree in the temperature of the perfusion.

Maximal temperature was related to TRNT, with shorter TRNT for higher temperatures. The relation of temperature to the occurrence of anastomotic leaks was ambivalent. However, the results indicate the possibility of a negative effect of increasing temperatures on the occurrence of anastomotic leaks.

Survival analysis did show an inverse relation between Tmax and post-operative survival both in univariate and multivariate analyses. Models including AUC instead of Tmax showed a similar inverse relationship between total temperature and survival.

Previously, in an animal model of PC a negative relation between survival and high temperature was also suggested as well ^19^.

The present study has several limitations. First it is not randomized. Hyperthermia is the standard for intraperitoneal chemoperfusion. The temperature was adjusted to the clinical status of the patients. Normothermic chemoperfusion was administered in patients with important comorbidity. This may cause a bias. However, survival is worse in patients treated with perfusate with a higher temperature although these were the patients with less comorbidity. Second, it is a retrospective study; therefore the data were not acquired in a standardized way which again is a possible source of biases. Third, due to limitations regarding the available data, not all potentially important variables (such as completeness of cytoreduction, lymph node status, whether or not the tumor was relapsing, etc.) could be included in the models.

Given these limitations, it is too early for a final conclusion on the relationship between hyperthermia and survival in the setting of HIPEC. However, the significantly worse survival in patients treated with hyperthermic intraperitoneal chemotherapy HIPEC raises important concerns about the safety of this method. Although, in selected patients, the results of HIPEC seems to be better than standard therapy ^2, 4^, treatment can possibly be further improved by reducing the temperature during the chemoperfusion to body temperature (i.e. 37–38°C). Future research on HIPEC should focus on two aspects of the treatment. First, randomized studies comparing normothermic to hyperthermic chemoperfusion are needed. Second, the causal mechanism of the worse survival in patients treated with hyperthermic chemoperfusion should be clarified.

## Consent

All data have been completely anonymised, without altering the scientific meaning of the analyses.

## Data availability

The data referenced by this article are under copyright with the following copyright statement: Copyright: © 2015 Verhulst J

Data associated with the article are available under the terms of the Creative Commons Zero "No rights reserved" data waiver (CC0 1.0 Public domain dedication). 

*Figshare:* Hyperthermic intraperitoneal chemoperfusion with high dose oxaliplatin: Influence of perfusion temperature on postoperative outcome and survival. doi: 10.6084/m9.figshare.730373 ^25^
