# Supplementary material for: Hyperthermic intraperitoneal chemoperfusion with high dose oxaliplatin: Influence of perfusion temperature on postoperative outcome and survival
Source: F1000Res. 2015 Oct 16;2:179. Originally published 2013 Sep 3. [Version 2] doi: 10.12688/f1000research.2-179.v2 (PMC4743150; doi:10.12688/f1000research.2-179.v2)
Supplement: Influence of perfusion temperature during hyperthermic intraperitoneal chemoperfusion on post-operative outcome and survival — Data shows clinical and biochemical features in patients with peritoneal carcinomatosis from different primary tumors. A list of abbreviations is included in the second file. [file f1000research-2-3893-s0000.tgz › 1138872]

AUC_30: area under the temperature curve over 30 minutes
Maxtemp_30: maximal temperature over 30 minutes
age: age at time of operation
sex: gender (0=male, 1=female)
length: length (in cm)
weight: weight (in kg)
optime: operation time (minutes)
TRNT: time to removal of nasogastric tube (days)
WBC_postop: leucocyte count one day after operation
AST_postop: AST level one day after operation
ALT_postop: ALT level one day after operation
GGT_postop: GGT level one day after operation
ICU: stay on intensive care unit (days)
hospstay: total hospital stay (days)
dead: 1= dead, 0 = censored
vitaldat: date of dead or censoring (days since January 1, 1960)
survival: days between operation and dead
anastomoses: number of anastomoses
diagnosis: 4=colorectal orign; 3= ovarian origin; 2= pseudomyxoma peritonei; else=1
operationdat= date of operation (days since January 1, 1960)
