# Supplementary material for: Hyperthermic intraperitoneal chemoperfusion with high dose oxaliplatin: Influence of perfusion temperature on postoperative outcome and survival
Source: F1000Res. 2015 Oct 16;2:179. Originally published 2013 Sep 3. [Version 2] doi: 10.12688/f1000research.2-179.v2 (PMC4743150; doi:10.12688/f1000research.2-179.v2)
Supplement: Peer review history

# Peer review: Referee response for version 2

**DOI:** 10.5256/f1000research.3893.r11383

**Published:** 2015-12-01

**Authors:** Pompiliu Piso

**Affiliations:** 1 University of Regensburg, Regensburg, Germany

The author has changed the manuscript according to the recommendation of one reviewer. This has improved the quality of the paper, however, I am still missing the most important data needed for survival analysis of patients with peritoneal metastases: completeness of cytoreduction and the amount of tumor expressed by the Peritoneal Cancer Index. There are many factors that also may play a role. The whole group was in the learning curve and during in this time, oncological results are poorer, as we know by now. The median survival of 23 months was a good as the in the Verwaals trial, however, this trial was performed many years before, still in the 5FU mono-therapy era. The average maximal temperature of 40.5 °C was rater moderate. In patients with higher AUC, this may be also related to the perfusion conditions, e.g. extended resection and ascites preoperatively, more peritoneal cavity and increased presentation to heat - but also higher morbidity and longer ICU stay. The Figure 13 is confusing. First, male and female have different survival in colorectal cancer, this is not new. Second, we have no information if the patients of same gender were matched pair, in particular with regard to completeness of cytoreduction and PCI. Only this way we could judge upon the role of hyperthermia.

I agree that prospective randomized trials are needed, to analyze the impact of hyperthermia alone in this setting. However, we first have to wait for the final results of the Prodige 7 trial to realize if hyperthermia + i.p. chemotherapy influence the prognosis the way many surgical oncologists believe now, based on the results of several patient series. The next step would be to randomize only hyperthermia, if the study shows an efficacy of HIPEC with regard to survival.

Nevertheless  hyperthermia was beneficial in many cancer treatment strategies and we know that the level of hyperthermia is important for the outcome. As required, the author may find some interesting ideas in the paper called "Hyperthermia adds to chemotherapy" written by Issels *et al *in Eur J Cancer 2008, 44:2456-2554.

So, yes the manuscript may stimulate to question the role of hyperthermia, however, the analysis have major deficits regarding the survival analysis and the reader can only speculate upon different effects. A bit unusual for me was the fact that the supervisor dissociated himself from any conclusions.

I have read this submission. I believe that I have an appropriate level of expertise to confirm that it is of an acceptable scientific standard, however I have significant reservations, as outlined above.

# Peer review: Referee response for version 2

**DOI:** 10.5256/f1000research.3893.r10840

**Published:** 2015-11-18

**Authors:** Shigeki Kusamura

**Affiliations:** 1 Department of Surgery, Fondazione IRCCS Itituto Nazionale Tumouri Milano, Milan, Italy

I do not agree that, on the basis of the available data, one can conclude that there may be a negative correlation between HIPEC temperature level and survival. In other words, the authors concluded that the higher the temperature, the shorter their survival.

To arrive to a such a conclusion one must conduct a comprehensive multivariable analysis using cox regression model. The only subset of patients in the cohort with sufficient sample size to attempt a minimal multivariate analysis is those affected by colorectal cancer (n=58). In the analysis conducted by the authors the main prognostic factors such as completeness of cytoreduction, PCI, age, type of perioperative systemic chemotherapy, lymph node status, presence of eventual liver metastasis, histologic differentiation were not considered as possible covariates, alongside the AUC of temperature (JCO Glehen 2004). Obviously the limited number of events (deaths) among the 58 cases would not have allowed the inclusion of all these covariates in the multivariable analysis but at least one such as PCI or completeness of cytoreduction should be considered.

I would accept the manuscript only if the authors decide to take out the inferences concerning survival from the study, as these conclusions have been obtained by imprecise and incorrect analysis.

I have read this submission. I believe that I have an appropriate level of expertise to confirm that it is of an acceptable scientific standard, however I have significant reservations, as outlined above.

# Peer review: Referee response for version 1

**DOI:** 10.5256/f1000research.867.r3009

**Published:** 2014-01-08

**Authors:** Shigeki Kusamura

**Affiliations:** 1 Department of Surgery, Fondazione IRCCS Itituto Nazionale Tumouri Milano, Milan, Italy

The author evaluated the correlation of temperature during HIPEC with short-term surgical and long term oncologic outcomes in patients affected by several types of peritoneal carcinomatosis. The idea is interesting, the paper reasonably well-written, and the analysis conducted meticulously.

The temperature was appraised using two parameters: Tmax and AUC.

- The first caveat of the paper is Tmax. If there is a possibility of tissue damage exerted by the heat, the negative effect depends not only on the level of temperature but also on the duration of the exposure to hyperthermic conditions. Under 43°C it is hard to observe significant tissue damage if the exposure does not last more than 30 minutes, according to experimental data. Therefore, in my opinion, this parameter is not suitable and unreliable to achieve the objectives of the study.
- The second weak point is the survival analysis. Important parameters such as PCI, completeness of cytoreduction, tumor grade, lymph node status (for colon cancer), and whether the tumor is primary or relapsing, the platinum sensitivity (for ovarian cancer) were not considered in the cox model. Moreover, in figure 13 the events occurred at the same time points from the surgery in all four subsets. Is that a coincidence? In the figure 14 groups 1 and 2 combined had 46 events (deaths). How could you explain if the total number of ovarian cases were 16 cases?
- The tables outlining logistic regression analysis could be better presented by changing the Estimates by Odds Ratio. Remember, OR=exp(B)=e ^estimate ^
  My advice is to reconsider the Tmax and survival analysis. Even though these parts contain the only significant correlations, I myself would take them out from the study. The correlations with short-term surgical outcome is sufficiently interesting to justify the publication.

I have read this submission. I believe that I have an appropriate level of expertise to confirm that it is of an acceptable scientific standard, however I have significant reservations, as outlined above.

# Peer review: Referee response for version 1

**DOI:** 10.5256/f1000research.867.r2235

**Published:** 2013-11-08

**Authors:** Pompiliu Piso

**Affiliations:** 1 University of Regensburg, Regensburg, Germany

This article is investigating the role of perfusion temperature on postoperative outcome and survival of patients with peritoneal carcinomatosis. The idea is interesting, however, the presented data does not support the conclusions.

The analysis was retrospective, the time interval very long, monocentric with a bias regarding surgeons, used technique and chemotherapy regime (mixed patients with HIPEC alone or plus intravenous 5FU).

The postoperative outcome is determined mainly by the extent of surgery and critical anastomosis and less by HIPEC itself. This has been shown by several published data. The TRNT is influenced by even more other factors. The causality relation between the maximal temperature and survival has not been demonstrated. Moreover, other significant prognostic factors e.g. peritoneal cancer index, completeness of cytoreduction, histologic subtype etc have not been evaluated. Out of any context, temperature may play in the statistical analysis a role but the clinical interpretation of the results should be very careful. For instance, who were the patients having maximal temperature during HIPEC: was this group having a high tumor load with a high Peritoneal Cancer Index?, a more difficult resection, were they co-morbid etc.

In my opinion, this manuscript is confusing rather than helpful. We know from other hyperthermia studies that efficacy is affected by every °C – this sounds more reasonable to me.

I have read this submission. I believe that I have an appropriate level of expertise to state that I do not consider it to be of an acceptable scientific standard, for reasons outlined above.

# Response

**Published:** 2014-01-13

**Authors:** Johanna Verhulst

**Affiliations:** Independent Researcher, Belgium

I am fully aware of the limitations due to the retrospective nature of the study. The intention of the study was to be a pilot study concerning the effects of hyperthermia in the context of HIPEC. The study does not claim to be able to draw any final conclusions on a causal relation between hyperthermia and post-operative survival. However, a negative correlation between those variables was observed. As it is not possible to make any final inference about this matter based on this study, further randomized studies should be conducted in order to confirm or disprove the results from this study.

As mentioned in the article, the patients treated with lower temperatures, were the patients with higher co-morbidity. Nevertheless, these patients had a longer survival in the studied population. Therefore, it could be expected that in a randomized study, the negative effect would be even bigger.

I would be grateful for references to articles studying the effects of hyperthermia on post-operative outcome.

# Peer review: Referee response for version 1

**DOI:** 10.5256/f1000research.867.r1679

**Published:** 2013-10-01

**Authors:** David L Morris

**Affiliations:** 1 Department of Surgery, University of New South Wales, NSW, Australia

This article certainly made me sit up and think. The finding of an adverse effect of T _max_ during HIPEC on long term survival is certainly very challenging. The science of the effects of hyperthermic intraperitoneal chemoperfusion is far from solid, but we have achieved previously unachievable survival results by using it. If this article is correct, we are harming our patients by using heat. I do not have the best statistical brain in the world, but the number of patients is small and for example there are only 21/138 patients with a maximum temperature of <39.5°C and only 10/138 with a temperature >41.5°C. The model used in figures 13 and 14 may well be valid but when there are only one or two patients with a T _max_ of 37°C it is not appropriate to plot a survival curve. The lactate data is interesting because it provides a clear illustration that a biologically important measure is being affected by temperature.

This paper creates more questions than answers but is clearly a stimulus to others to examine their data.

I have read this submission. I believe that I have an appropriate level of expertise to confirm that it is of an acceptable scientific standard.
